# Development and Validation of Predictive Models for Non-Adherence to Antihypertensive Medication

**DOI:** 10.3390/medicina61071313

**Published:** 2025-07-21

**Authors:** Cristian Daniel Marineci, Andrei Valeanu, Cornel Chiriță, Simona Negreș, Claudiu Stoicescu, Valentin Chioncel

**Affiliations:** 1 Department of Pharmacology and Clinical Pharmacy, Faculty of Pharmacy, Carol Davila University of Medicine and Pharmacy, 020956 Bucharest, Romania; daniel.marineci@umfcd.ro (C.D.M.); cornel.chirita@umfcd.ro (C.C.); simona.negres@umfcd.ro (S.N.); 2 Department of Cardiology, Faculty of Medicine, Carol Davila University of Medicine and Pharmacy, 020021 Bucharest, Romania; claudiu.stoicescu@umfcd.ro (C.S.); valentin.chioncel@umfcd.ro (V.C.); 3 Cardiology and Cardiovascular Surgery Department, University Emergency Hospital Bucharest, 050098 Bucharest, Romania; 4 Department of Cardiology, Emergency Clinical Hospital Prof. Dr. Bagdasar Arseni, 041915 Bucharest, Romania

**Keywords:** medication adherence, antihypertensive therapy, machine learning predictive models, permutation feature importance analysis, probability-based adherence stratification

## Abstract

*Background and Objectives*: Investigating the adherence to antihypertensive medication and identifying patients with low adherence allows targeted interventions to improve therapeutic outcomes. Artificial intelligence (AI) offers advanced tools for analyzing medication adherence data. This study aimed to develop and validate several predictive models for non-adherence, using patient-reported data collected via a structured questionnaire. *Materials and Methods*: A cross-sectional, multi-center study was conducted on 3095 hypertensive patients from community pharmacies. A structured questionnaire was administered, collecting data on sociodemographic factors, medical history, self-monitoring behaviors, and informational exposure, alongside medication adherence measured using the Romanian-translated and validated ARMS (Adherence to Refills and Medications Scale). Five machine learning models were developed to predict non-adherence, defined by ARMS quartile-based thresholds. The models included Logistic Regression, Random Forest, and boosting algorithms (CatBoost, LightGBM, and XGBoost). Models were evaluated based on their ability to stratify patients according to adherence risk. *Results*: A total of 79.13% of respondents had an ARMS Score ≥ 15, indicating a high prevalence of suboptimal adherence. Better adherence was statistically associated (adjusted for age and sex) with more frequent blood pressure self-monitoring, a reduced salt intake, fewer daily supplements, more frequent reading of medication leaflets, and the receipt of specific information from pharmacists. Among the ML models, CatBoost achieved the highest ROC AUC Scores across the non-adherence classifications, although none exceeded 0.75. *Conclusions*: Several machine learning models were developed and validated to estimate levels of medication non-adherence. While the performance was moderate, the results demonstrate the potential of AI in identifying and stratifying patients by adherence profiles. Notably, to our knowledge, this study represents the first application of permutation and SHapley Additive exPlanations feature importance in combination with probability-based adherence stratification, offering a novel framework for predictive adherence modelling.

## 1. Introduction

Non-adherence to antihypertensive medication is a major barrier to effective blood pressure control, and it refers to a patient’s failure to follow the prescribed treatment regimen as recommended by a healthcare provider. Non-adherence may involve delays in starting therapy, missing doses, incorrect timing or dosage, or prematurely discontinuing treatment [1]. It can be intentional (e.g., due to concerns about side effects or cost) or unintentional (e.g., forgetfulness or misunderstanding instructions) [2]. 

Non-adherence to medication leads to poorer clinical outcomes, increased patient suffering, and higher morbidity and mortality. It also results in greater healthcare-related costs, reduced productivity, and a lower quality of life [3]. A recent meta-analysis of 12 studies involving over 2 million patients found that poor adherence to antihypertensive therapy is significantly associated with increased all-cause mortality and cardiovascular mortality. The risk was notably higher among patients undergoing long-term treatment [4].

Studies report varying levels of adherence to antihypertensive medication. Cross-sectional research conducted across diverse global populations has found adherence rates ranging from 27.9% to 85% [5]. This wide variability likely reflects differences in patient demographics, healthcare systems, cultural beliefs, and the methods used to measure adherence. The heterogeneity across studies highlights the need for standardized assessment tools and further investigation into context-specific barriers and facilitators of adherence. Identifying these factors is essential for designing targeted interventions to improve long-term treatment outcomes in hypertension management.

In the management of hypertension, medication adherence is a critical determinant of therapeutic success, yet it remains suboptimal in many cases due to the asymptomatic nature of the condition. Factors such as an older age, low health literacy, cognitive decline, and polypharmacy further complicate adherence, particularly in elderly patients who are the primary users of antihypertensive drugs. Complex therapeutic regimens have been consistently associated with lower adherence rates. Beliefs and attitudes toward medication also play a vital role; patients who understand the risks of uncontrolled hypertension (e.g., stroke, myocardial infarction) and believe in the efficacy of treatment are more likely to adhere, while those concerned about side effects or long-term dependence are more likely to interrupt therapy. A strong, trust-based relationship between the patient and the physician, effective communication, and the continuity of care have all been associated with better adherence outcomes. System-level barriers, such as high out-of-pocket costs or limited access to healthcare services, can significantly hinder adherence. Moreover, social and familial support can enhance adherence by promoting routines and reinforcing the importance of health behaviors [6,7,8]. The optimal management of arterial hypertension requires educating the patient about adherence to the antihypertensive treatment, including diuretics, lifestyle modifications, and monitoring for adverse effects [9].

Machine learning (ML) algorithms are capable of processing and integrating diverse data types, including the prescription history, insurance claims, patient demographic information, and real-time data from medication adherence monitoring systems. Through this approach, ML models can uncover complex associations and behavioral patterns that are often undetectable using traditional analytical methods. By learning from these patterns, ML algorithms can accurately estimate patient adherence to prescribed treatments [10]. These predictive models enable the stratification of patients based on their risk of non-adherence, thus supporting the implementation of personalized and proactive interventions. In this way, ML-driven technologies play a critical role in enhancing the quality of the healthcare delivery and mitigating the negative consequences of non-adherence on both patient outcomes and healthcare system costs [11].

Given the significant impact of non-adherence on hypertension management and the limitations of current predictive approaches [12], our study aims to move beyond a descriptive analysis and toward an actionable prediction. After outlining the multifactorial nature of non-adherence, we advance the discussion by constructing and validating machine learning models designed to identify patients at risk of poor adherence based on a real-world dataset of 3095 ARMS questionnaires. The models were mainly built on sociodemographic and behavioral data, as collected through a structured, non-clinical questionnaire, reflecting a pragmatic approach applicable in settings where pharmacological or clinical data may be unavailable.

## 2. Materials and Methods

### 2.1. Study Design

The questionnaire collected data on several dimensions relevant to this study. Sociodemographic characteristics included the participant’s age (as a continuous variable, in years), sex (male or female), living situation (alone vs. with family/partner), and highest level of formal education completed, (categorized as middle school, high school, and university degree or higher). Medical and pharmaceutical aspects focused on the patient’s co-existing health conditions and the number of medications and dietary supplements used daily. Next item addressed the frequency of self-monitoring blood pressure; the responses (scored 5 for “daily”, 4 for “several times per week”, 3 for “once per week”, 2 for “only when feeling unwell”, and 1 “only at the physician’s office”) generated an ordinal variable, with higher values indicating less frequent or absent self-monitoring behaviors.

The most recent measured value of their blood pressure, based on the question “*What was your blood pressure at the last measurement?*”, assessed the participant’s recent level of blood pressure control, in alignment with commonly used clinical thresholds for hypertension management. The variable was treated as binary, with values coded 1 for controlled blood pressure (<140/90 mmHg) and 0 for uncontrolled blood pressure (≥140/90 mmHg).

Salt-related dietary behavior was evaluated through the question “*Do you add salt to your food before tasting it?*”, while the level of patient engagement with pharmacological information was assessed by asking “*Do you read the information leaflets for your prescribed medications?*” Both questions used the same four-point Likert-type response scale (“never”, “sometimes”, “most of the time”, and “always”, coded accordingly from 1 to 4, with higher scores indicating more frequent behavior).

To explore the nature of patient–medication interaction, two items assessed the type of information that patients found most relevant and the extent of counseling received from pharmacists at the time of dispensing. Participants were asked “*What information from the medication leaflet interests you the most?*”, with predefined multiple-choice options: dosage, route of administration, and duration of treatment.

A related question assessed pharmacist-delivered counseling: “*When receiving a prescribed medication, what kind of information does the pharmacist provide you with?*” Response options included the following: dosage, route of administration, duration of treatment, and potential adverse drug reactions. Both items were analyzed descriptively to determine which aspects of drug therapy patients prioritize and to evaluate the completeness of pharmaceutical counseling.

Medication adherence and consistency in refilling prescriptions were assessed using the 12-question ARMS (Adherence to Refills and Medications Scale) questionnaire [13]. The ARMS captures both intentional and unintentional non-adherence, making it a valuable tool for identifying patients at risk and informing tailored interventions.

ARMS is a validated self-report instrument designed to assess medication adherence, particularly in populations with chronic conditions, that evaluates two key dimensions of adherence: medication-taking behavior (e.g., forgetting to take a dose, taking medications late) and prescription refill behavior (e.g., running out of medication before refilling, delays in obtaining refills). Each item is scored on a 4-point Likert scale (1 “never”, 2 “sometimes”, 3 “most of the time”, 4 “always”). The total ARMS Score ranges from 12 (reflecting perfect adherence) to 48, higher scores reflecting progressively lower levels of adherence [13,14,15]. While no universal cut-off exists, some studies use sample-based thresholds (e.g., median or upper quartile) to classify participants into “more adherent” vs. “less adherent” groups [16]. In this study, adherence levels were interpreted based on the distribution of scores in the sample, and quartile-based classification was applied for analysis.

Permission to translate and use the ARMS tool for research purposes was obtained from its original developers at Emory University, Atlanta, GA, USA, led by Sunil Kripalani [13]. Two independently translated Romanian versions were harmonized and then back-translated into English. The resulting versions were compared with the original to select a Romanian form that was both faithful to the source and easy for patients to understand. Validation of the Romanian translation, based on 100 completed questionnaires, demonstrated good internal consistency, with a Cronbach’s alpha of 0.90.

The interviews were conducted in community pharmacies across Bucharest between April and June 2019. Pharmacists—mostly clinical pharmacy residents—identified potential participants among hypertensive patients. After verifying the inclusion criteria (adult patients diagnosed with hypertension and on antihypertensive therapy for at least three months), participants were informed about the study and asked for their verbal informed consent. Patients who voluntarily agreed to participate were subsequently administered the questionnaire. The pharmacist read the questions aloud and recorded the participants’ responses.

### 2.2. Preliminary Statistical Analysis

The preliminary statistical analysis was undertaken in Python Programming Language, version 3.9.2, by the use of scipy and pingouin packages. Since the ARMS score did not follow a normal distribution (as assessed by the Shapiro–Wilk test), non-parametric tests were applied to assess differences across groups defined by categorical variables. Specifically, the Kruskal–Wallis H test was used [17,18].

In addition, in order to characterize the obtained 3095-patient dataset with regard to the interrelation between the collected variables and the total ARMS Score, an age- and sex-adjusted Spearman correlation was computed between both binary and continuous parameters and the total ARMS Score, as well as on three binary outcomes considered by setting specific thresholds. More precisely, due to the fact that ARMS Score was found to follow a non-Gaussian distribution, the three quartiles were chosen as specific non-adherence thresholds (Q1 = 15, Q2 = 18, Q3 = 22); in each case, the ARMS Scores of at least the quartile value were set to belong to class 1 (less adherent patient group), while values lower than that of the quartile were included in class 0 (more adherent patient group) [17,18,19]. The reasoning behind the adjusted correlation analysis was to extract important associations between the most relevant modifiable influencers of adherence and the ARMS Score, highlighting their potential role as predictive variables.

### 2.3. The Development and Validation of Non-Adherence Prediction Models

Based on the variables which were analyzed during the adjusted Spearman correlation computation (a total of 28 predictive variables), five machine learning models were developed and validated based on their ability to perform patient stratification based on the three quartile-based non-adherence outcomes. More specifically, for each of the three outcomes, Logistic Regression, boosting algorithms (CatBoost, Light Gradient Boosting Machines (Light GBM), and Extreme Gradient Boosting (XGBoost)), and Random Forest machine learning models were built and validated in a 5-time repeated 5-fold nested cross-validation (CV) manner. For each fold, the training set was used for Bayesian hyperparameter tuning through Optuna package (25 trials were used for each model and the Receiver Operating Characteristic Curve (ROC AUC) Score was used as optimization metric), and the models tuned on the training set through a 3-fold cross-validation were evaluated afterwards on the test set. The ROC AUC Score was the main validation metric, since it estimated the reliability of the predicted probabilities of belonging to class 1, while the Matthews Correlation Coefficient (MCC) was the most relevant metric for assessing the raw classification ability, followed by precision, recall, and accuracy. In addition, the predictive importance of each feature was estimated through the permutation feature importance method, by computing the improvement in ROC AUC Score obtained by each predictive variable on the test set. In addition, for the best performing model in terms of ROC AUC Score (CatBoost), the SHapley Additive exPlanations (SHAP) feature importance was also computed, by taking into account the final model, and trained on the entire dataset, according to the shap package recommendations. The machine learning models’ development and validation, as well as the permutation feature importance, were implemented by the use of the following Python packages: scikit-learn, catboost, lightgbm, xgboost, and shap [18,20,21,22,23,24].

## 3. Results

### 3.1. Preliminary Statistical Analysis

**Figure 1:**
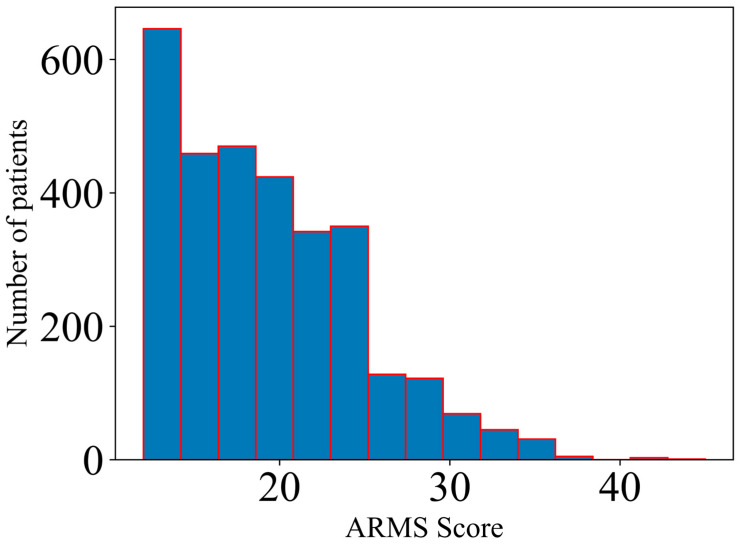
The distribution of the adherence scale (ARMS Score).

For an enhanced visualization of the adherence scale, Figure 1 illustrates the distribution of the total ARMS Score (Adherence to Refills and Medications Scale) through a histogram plot with 15 bins. Table 1 presents the results obtained for the non-parametric tests (difference across groups in terms of ARMS Score), while Table 2 presents the results obtained within the adjusted correlation analysis. The Spearman correlation coefficient is given, along with the *p* value for the statistical significance. For the binary outcomes, the percentage of patients with an ARMS Score at least equal to the specific quartile value is also given. The significance threshold was set at <0.05 for both analyses.

### 3.2. Validation Results of the Machine Learning Models

Table 3 presents the results obtained during the validation of the five machine learning algorithms, while Table 4, Table 5 and Table 6 highlight the importance of the predictive variables, computed through the permutation feature importance method. For each predictive variable, algorithm, and quartile-based outcome, the percentual improvement in the ROC AUC Score out of the total improvement is given. In addition, Table 7 presents the importance of the predictive variables, computed through the SHapley Additive exPlanations (SHAP) method (permutation algorithm type, automatically chosen based on the data) for CatBoost and all quartile-based outcomes; for each variable, the percentual contribution out of the total mean absolute shap values for the entire dataset is given.

From the perspective of precision, all models performed best at the Q1 threshold (ARMS >15), with values ranging from 0.863 to 0.873, indicating a strong ability to correctly identify patients at a moderate risk of non-adherence. The precision gradually declined with higher thresholds, reaching values around 0.46–0.47 for an ARMS Score > 22, suggesting an increased difficulty in detecting severe non-adherence, likely due to the class imbalance and fewer positive cases. In contrast, the Matthews Correlation Coefficient showed an opposite trend: scores were higher for an ARMS Score > 22 (up to 0.32), indicating a better alignment between model predictions and actual outcomes in severe non-adherence cases, despite their rarity. The Matthews Correlation Coefficient (MCC) thus offered a more balanced perspective on the raw classification performance, which is especially valuable in the presence of imbalanced classes.

The ROC AUC further supported these observations, reflecting the overall discriminatory power of the models. All AUC scores were above 0.70, confirming a solid performance in distinguishing between adherent and non-adherent patients. Among the tested algorithms, CatBoost achieved the highest scores, ranging from 0.718 to 0.734 across the three thresholds. This suggests that CatBoost effectively captures complex relationships between predictors and adherence levels, providing stable and differentiated predictions across the full spectrum of severity. Overall, the multi-metric evaluation confirms that the models are most effective in identifying moderate-risk patients but shows a better alignment with true outcomes in severe non-adherence, with CatBoost standing out for its balanced and superior performance.

## 4. Discussion

In the current study, several machine learning models were developed and validated based on their ability to estimate the non-adherence status based on specific ARMS (Adherence to Refills and Medications Scale) quartile-based thresholds (Q1 = 15, Q2 = 18, Q3 = 22), taking into account various sociodemographic and clinical characteristics from 3095 patients as predictive features.

The first step of the analysis involved evaluating the differences across groups in terms of ARMS Scores and computing age- and sex-adjusted Spearman’s correlation coefficients between the included variables and the non-adherence status, quantified both by the total ARMS Score and by the three binary outcomes which were further used for building the machine learning algorithms. The results obtained in terms of the inter-group comparison for the categorical variables (Table 1) yielded statistically significant differences for the majority of the variables; in terms of the level of significance, it should be noted that *p* values lower than 0.001 were obtained for family support; the level of education; respiratory, rheumatic, and central nervous system diseases; blood pressure (BP) self-monitoring; and the frequency of adding salt in food and of reading patient information leaflets (PILs), as well as for the majority of the PIL- and pharmacist-related information, therefore implying that the majority of the variables included in this study might be used for patient stratification in terms of adherence levels.

On the other hand, the results obtained in terms of adjusted Spearman’s correlation coefficients (Table 2) highlighted statistically significant (*p* < 0.05) associations between the majority of the predictive variables and the adherence status of the patients. More specifically, for the total ARMS Score, the highest positive correlations were obtained for the frequency of adding salt in food (r = 0.254), followed by a high blood pressure during self-monitoring (r = 0.215) and the number of daily administered supplements (r = 0.106). On the other hand, the most significant negative correlations were obtained for the frequency of reading patient information leaflets (r = −0.239), followed by the normal blood pressure during self-monitoring (r = −0.214) and the frequency of blood pressure self-monitoring (r = −0.184). The binary quartile-based outcomes showed similar patterns with regard to their age- and sex-adjusted associations with the predictive variables. For example, the number of daily administered supplements had identical values of Spearman’s correlation coefficients for both the Q1- (the status of ARMS ≥ 15) and Q2- (the status of ARMS ≥ 18) based outcomes (r = 0.118), while for the Q3-based binary outcome (corresponding to the status of ARMS ≥ 22, hence less adherent patients), a smaller and non-significant correlation coefficient was obtained (r = 0.033, *p* = 0.068). The influence of the blood pressure self-monitoring and the results of the monitoring (whether normal or high blood pressure) were similar for all three binary outcomes but slightly more pronounced for Q2 and Q3 thresholds (for example the normal blood pressure during self-monitoring yielded Spearman’s correlation coefficients of −0.139 for Q1, −0.189 for Q2, and −0.188 for Q3). A similar pattern was observed for the frequency of adding salt in food, while for the frequency of reading patient information leaflets, the associations were more similar between all three binary outcomes, with a slightly stronger association with the Q3-based outcome (r = −0.177, −0.17, and −0.207, respectively, for Q1, Q2, and Q3). Moreover, with regard to the type of information that the patients received from the leaflets, the adverse drug reactions had the highest associations, even though to a lower magnitude than for the already mentioned variables, while the method of administration of the pharmacist-related counselling information most highly correlated to all binary non-adherence measures (r = −0.084, −0.066, and −0.065, respectively, for Q1, Q2, and Q3). Overall, blood pressure self-monitoring, a lower frequency of adding salt in food, a lower number of daily administered supplements, reading patient information leaflets to a higher degree, and the specific information received from the community pharmacists were found to have small to moderate, but statistically significant, age- and sex-adjusted associations with being more adherent to the drug therapy—findings already highlighted in various studies from different countries [25,26,27,28,29].

More specifically, with regard to the influence of various factors on adherence levels, it is worth mentioning that multiple studies confirm that higher education levels are strongly associated with a better adherence to antihypertensive treatment. Zyoud et al. (2013) found that university-educated patients showed significantly higher adherence and a better health-related quality of life compared to less educated individuals [30]. Similarly, Hussein et al. (2020) reported that patients with a secondary or higher education were more adherent than those with only a primary education [31]. These results highlight education as a key predictor of adherence, supporting our study’s findings, where the higher the education level, the lower the ARMS adherence score, indicating a better adherence (Spearman correlation coefficient ρ = −0.168, *p* < 0.001) [32].

In general, the greater the number of medications taken per day and the higher the dosing frequency, the lower the adherence [33,34]. Our research reveals similar findings: the more complex the medication regimen, the lower the adherence to treatment. Over 40% of respondents did not regularly self-monitor their blood pressure, with 17.3% measuring it only during doctor visits and 24.7% doing so only when they felt unwell. These individuals had higher ARMS adherence scores—indicating lower adherence—and poorer blood pressure control compared to those who monitored their blood pressure regularly, either daily or at least weekly. This finding aligns with other studies showing that regular blood pressure monitoring is positively associated with a better adherence to antihypertensive medication [35].

In addition, a belief in the necessity and effectiveness of medications increases adherence [34]. In our study, knowledge about medications—as indicated by the frequency with which patients read the patient information leaflets [36]—was associated with a higher adherence to medication and refills. Adverse drug reactions, or the fear of such reactions, reduce treatment adherence. Studies show that non-adherent patients generally experience more frequent or more severe side effects compared to those who are adherent [37]. This highlights the importance of managing adverse drug reactions and educating patients on how to recognize them early. The fear of side effects and concerns about therapy are also associated with decreased adherence [38]. It is noteworthy that, in our investigation, the section of the medication leaflet that attracted the most interest among patients was the one concerning adverse effects.

The adjusted correlation analysis was followed by the development of several predictive algorithms, with the main aim of predicting, based on both the probability estimation and raw classification, the non-adherence status, defined based on the ARMS status of at least Q1 (ARMS = 15), Q2 (ARMS = 18), or Q3 (ARMS = 22), respectively. The validation results, presented in Table 3, yielded the highest results in terms of ROC AUC Scores for CatBoost for all three outcomes (ROC AUC = 0.728 for Q1, 0.718 for Q2, and 0.734 for Q3). The ROC AUC Score, which was the indicator for estimating the reliability of the predicted probabilities of belonging to class 1 (the non-adherent group for all three binary outcomes), was considered the most important machine learning validation indicator, since its use as an optimization metric ensures an enhanced stratification of patients based on their adherence levels. The obtained values were higher than 0.7 for all five machine learning models (but were the highest for the CatBoost model, an important type of gradient boosting algorithm, which is designed to reduce bias and improve handling of categorical variables [21]) and were considered satisfactory, given the moderate sample size of the included dataset (3095 patients) and the relatively low total number of included predictors (28 variables).

With regard to other studies which aimed at implementing machine learning for estimating patients’ adherence, Lucas et al. used Electronic Health Records data from more than 100,000 patients to estimate the adherence to statin treatment through Random Forest. The c-statistic obtained during the cross-validation process, which estimated the ability of the model to rank individuals based on statin adherence levels, was 0.736, which was almost identical to the optimal ROC AUC Score obtained for the Q3-based outcome (0.734), even though the current study used a significantly lower number of patients and potential predictors; nevertheless, the study conducted by Lucas et al. focused on a specific subtype of drug therapy adherence, while in our analysis, we used a broader, even though much smaller, patient cohort with regard to the medication types [39]. In another research project that involved the use of machine learning for estimating adherence, Karanasiou et al. implemented various algorithms (such as Support Vector Machines, Multi-Layer Perceptron, Random Forest, and Naïve Bayes) to classify 90 heart failure patients based on their global adherence and medication adherence [40]. However, despite using various types of machine learning models, the study only performed raw classification and only reported accuracy as a performance metric, which might be biased, especially in the case of an imbalanced class task [41]. Moreover, it is worth mentioning that in our study we additionally reported the accuracy, precision, recall, and Matthews Correlation Coefficient (MCC); the MCC is frequently considered a more robust and reliable classification evaluation metric, even when comparing it to the more classical F1 Score; for the Q1-based outcome we obtained an optimal MCC of 0.279 by using XGBoost; in addition, a maximum MCC of 0.320 was computed for the Q2-based outcome with the CatBoost algorithm, while for the Q3-based outcome the best MCC was 0.317 when using the XGBoost model. These values were considered satisfactory, especially given the fact that the MCC can obtain values from a minimum of −1 and a maximum of 1, which is different than the 0–1 scale of the traditional classification evaluation indicators [42]. The precision and recall showed relatively balanced values, but precision had a higher variability, with a minimum of 0.445 (for the Q3-based outcome) and a maximum of 0.874 (for the Q1-based outcome with Logistic Regression); on the other hand, the computation of the recall yielded values between 0.611 (for the Q3-based outcome) and 0.773 (for the Q1-based outcome with XGBoost). Therefore, the focus on minimizing false negative values was more robust, regardless of the adherence threshold.

In addition, other similar studies which implemented machine learning in patient adherence estimations involved the use of the LASSO regression on medical claims data from more than 10,000 patients with myocardial infarction, with a focus on statin therapy [43], while Galozy et al. implemented tree-, Logistic Regression-, and K-nearest neighbors-based algorithms for predicting the medication refill adherence from Electronic Health Records data; in addition, Lee et al. used Logistic Regression and Support Vector Machines for estimating adherence (quantified through the Morisky Scale) in 293 patients with chronic diseases, with the AUC being the main measure for validating the classification performance [44,45]. In our study, we mainly focused on tree-based algorithms due to the size and structure of the dataset (relatively small sample size, few continuous variables, and cases with imbalanced classes). Moreover, it should be mentioned that the studies which implemented various machine learning algorithms for adherence predictions did not use an uniform adherence definition or scale, which makes performing a standardized comparison to our study more difficult [1,10,46,47].

One of the main advantages of the current study was the computation of the importance of each predictive variable in the estimation of the probability of belonging to the non-adherent group for each three quartile-based binary outcomes. While it was not the first time that the permutation feature importance was used to assess the explainability of the predictors in adherence estimations (for example, Choe et al. used it when estimating adherence to physical activity guidelines [48]; Mirzadeh et al. also computed the contribution of predicting variables in machine learning models for estimating adherence to drug therapy in patients at risk of atherosclerotic cardiovascular disease, even though this was not through permutation feature importance [49]), to our knowledge it is the first time that the permutation feature importance has been used in combination with a stratification based on a probability estimation. The results (presented in Table 4, Table 5 and Table 6) highlight the percentage of the ROC AUC Score improvement on the test set for each feature when comparing it to the improvement in ROC AUC Score for all features combined. The obtained percentages followed a similar pattern to the one observed in the adjusted correlation analysis. Hence, it is worth mentioning that the frequency of adding salt in food, of reading patient information leaflets, and of blood pressure self-monitoring were the most informative features for all three outcomes, with a percentual importance of at least 5% in all cases and a maximum importance of over 25–30%, as observed by the CatBoost model (which was the best model in terms of the probability estimation, as validated through the ROC AUC Score). Other important predictors in terms of the ROC AUC improvement were the number of daily administered supplements (only for Q1- and Q2-based outcomes), the level of education (especially for the Q3-based outcome, corresponding to identifying the most non-adherent patients), and the pharmacist-related counselling method of administration (especially for the Q1-based outcome). In addition, in order to quantify the clinical utility of the predictive variables in the final, best performing model in terms of ROC AUC Scores (CatBoost) and to evaluate the interpretability of the ML algorithm, a SHapley Additive exPlanations (SHAP) analysis was also undertaken, based on the whole dataset; the results are presented in Table 7. In this regard, it is worth mentioning that the contributions of each variable followed similar patterns to the ones observed for the permutation feature importance. The highest contributions were observed for all quartile-based thresholds for the frequency of adding salt in food (11.02% for an ARMS Score of 15, 15.88% for an ARMS Score of 18, and 20.52% for an ARMS Score of 22) and of reading PILs (11% for an ARMS Score of 15, 8.28% for an ARMS Score of 18, and 13.55% for an ARMS Score of 22), while a high BP during self-monitoring had almost a double contribution for the highest quartile-based threshold (Q1—ARMS Score ≥ 22) when compared to the two other thresholds (approximately 10%, compared to 5%), and the number of daily administered supplements contributed the most for the first quartile, followed by the second quartile. Therefore, given the similar trends that were observed by implementing two different feature importance methods, this study presents an important justification for considering specific predictors for explaining, in a clinically relevant manner, the levels of the non-adherence of Romanian patients taking antihypertensive medication. Overall, it is worth mentioning that the blood pressure self-monitoring, the frequency of adding salt in food, and the frequency of patient information leaflet reading helped in stratifying patients regardless of their adherence levels, while the use of supplements, the level of education, and specific pharmacist counselling might help in discerning specific adherence levels only, which reflects the personalized and non-linear characteristics of medication adherence with regard to its associations with relevant influencing factors [29].

One important limitation of this study is the exclusion of pharmacological variables known to influence adherence, such as the antihypertensive drug class, treatment complexity (monotherapy vs. combination therapy), and packaging features. This exclusion was a deliberate design choice aligned with this study’s main objective: to assess the feasibility of predicting antihypertensive adherence using easily obtainable, non-clinical variables. The predictor set was defined during the questionnaire development, with a focus on sociodemographic and behavioral factors that are modifiable and relevant for educational and public health interventions. Although this approach limited the scope of the models, it reflects a pragmatic and scalable design. Moreover, although some differences in the adherence between antihypertensive drug classes have been reported, the overall adherence remains suboptimal across all classes [50], emphasizing the relevance of the behavioral dimensions targeted in this study. Future research could expand on our findings by integrating clinical and pharmacological data to enhance the predictive performance and broaden applicability.

In conclusion, in the current study several predictive models were developed and validated based on their ability to estimate the medication non-adherence, as quantified though various thresholds. The validation results through the ROC AUC Score show the potential of machine learning to identify and stratify patients based on their adherence levels. While the ROC AUC values of 0.70–0.74 indicate a moderate predictive performance, the models were developed using only non-clinical, self-reported data, which makes them highly feasible for use in real-world settings. Their practical utility lies in serving as low-cost, scalable tools for the early identification of patients at risk of non-adherence, particularly in primary care or public health contexts where the access to clinical data may be limited. Despite the inherent disadvantages of the current study (relatively small patient cohort, when compared to other studies; lack of universally accepted standardized adherence scales; lack of external validation for the developed machine learning models), such results, after being included along with the optimized prediction algorithms in an online platform, might help to develop personalized intervention recommendations related to the adherence to medication, with a consequent improvement in the control of chronic illnesses and the quality of life.

## 5. Conclusions

This current study developed and validated predictive models for estimating the non-adherence levels in hypertensive patients. This research was undertaken by collecting relevant information from 3095 patients and defining adherence through the ARMS Score, followed by building the machine learning algorithms aimed at estimating the non-adherence levels, defined by three quartile-based thresholds. The results yielded ROC AUC Scores of over 0.7, which were optimal for the CatBoost model with a reasonable ability to stratify patients based on their probability of non-adherence, while the variable of the highest importance for the predictive ability was obtained from the level of education, number of daily administered supplements, blood pressure self-monitoring, and the frequency of adding salt in food and of reading patient information leaflets. Future studies must aim to optimize the ability of the machine learning algorithms to stratifying the patients and develop an online platform which could be used by physicians and pharmacists for optimizing adherence levels and improving treatment outcomes.

## Figures and Tables

**Table 1:** The results for the non-parametric tests regarding the difference across groups in terms of the ARMS Score.

Variable	Group	Percentage or Median (IQR)	ARMS Score Median (IQR)	*p* Value (Kruskal–Wallis)
Age (years)		63 (19)	18 (7)	-
Sex	Female	55.8%	18 (7)	0.008
	Male	44.2%	19 (8)	
Family support	Living alone	25.72%	19 (8)	<0.001
	Living with family/partner	74.28%	18 (7)	
Level of education	Middle school or lower	17.77%	20 (8)	<0.001
	High school	43.52%	18 (7)	
	University degree or higher	38.71%	18 (6)	
Number of daily administered drugs		4 (3)	18 (7)	-
Number of daily administered supplements		1 (2)	18 (7)	-
Cardiovascular diseases (other than HBP)	No	31.34%	19 (8)	0.105; NS
	Yes	68.66%	18 (7)	
Respiratory diseases	No	85.62%	18 (7)	<0.001
	Yes	14.38%	19 (7)	
Diabetes	No	76.28%	18 (7)	0.398; NS
	Yes	23.72%	19 (7)	
Rheumatic diseases	No	72.86%	18 (7)	<0.001
	Yes	27.14%	19 (7)	
Central nervous system diseases	No	86.11%	18 (7)	<0.001
	Yes	13.89%	19 (7)	
Gastrointestinal diseases	No	72.41%	18 (7)	0.032
	Yes	27.59%	19 (8)	
Endocrine diseases	No	86.43%	18 (7)	0.886; NS
	Yes	13.57%	18 (7)	
Neoplasm	No	96.7%	18 (7)	0.004
	Yes	3.3%	17 (6)	
Other diseases	No	79.97%	18 (7)	0.025
	Yes	20.03%	19 (8)	
BP self-monitoring	Only at the physician’s office	17.32%	19 (8)	<0.001
	Only when feeling unwell	24.65%	19 (7)	
	Once per week	15.12%	18 (7)	
	Several times per week	26.2%	18 (6)	
	Daily	16.7%	17 (6)	
Normal BP during self-monitoring	No	36.28%	20 (7)	<0.001
	Yes	63.72%	17 (7)	
Frequency of adding salt in food	Never	33.89%	17 (6)	<0.001
	Sometimes	37.67%	19 (8)	
	Most of the time	18.93%	20 (7)	
	Always	9.5%	20 (9)	
Frequency of reading PIL	Never	14.8%	20 (9)	<0.001
	Sometimes	38.77%	19 (7)	
	Most of the time	23.49%	18 (6)	
	Always	22.93%	16 (7)	
Dosage (element of interest from PIL)	No	66.04%	19 (8)	<0.001
	Yes	33.96%	17 (7)	
Method of administration (element of interest from PIL)	No	40.06%	19 (8)	0.01
	Yes	59.94%	18 (7)	
Treatment duration (element of interest from PIL)	No	75.67%	18 (7)	0.139; NS
	Yes	24.33%	18 (7)	
Adverse drug reactions (element of interest from PIL)	No	38.64%	19 (7)	<0.001
	Yes	61.36%	18 (7)	
Dosage (pharmacist-related counselling)	No	37.87%	19 (8)	<0.001
	Yes	62.13%	18 (7)	
Method of administration (pharmacist-related counselling)	No	12.18%	20 (7)	<0.001
	Yes	87.82%	18 (7)	
Treatment duration (pharmacist-related counselling)	No	58.64%	18 (7)	0.314; NS
	Yes	41.36%	19 (7)	
Adverse drug reactions (pharmacist-related counselling)	No	60.23%	19 (8)	0.027
Adverse drug reactions (pharmacist-related counselling)	Yes	39.77%	18 (7)	

Legend: HBP = high blood pressure; PIL = patient information leaflets; and NS = non-significant.

**Table 2:** The age- and sex-adjusted Spearman Correlation Coefficient computed between the included variables and the ARMS Score (both continuous- and quartile-based binary outcomes).

Variable	ARMS (Continuous)	Q1 (ARMS ≥ 15: 79.13% of Patients)	Q2 (ARMS ≥ 18: 56.80% of Patients)	Q3 (ARMS ≥ 22: 29.21% of Patients)
Family support	−0.092 (*p* < 0.001)	−0.041 (*p* = 0.023)	−0.069 (*p* < 0.001)	−0.078 (*p* < 0.001)
Level of education	−0.168 (*p* < 0.001)	−0.1 (*p* < 0.001)	−0.118 (*p* < 0.001)	−0.154 (*p* < 0.001)
Number of daily administered drugs	0.004 (*p* = 0.822; NS)	0.015 (*p* = 0.409; NS)	0.018 (*p* = 0.316; NS)	−0.023 (*p* = 0.197; NS)
Number of daily administered supplements	0.106 (*p* < 0.001)	0.118 (*p* < 0.001)	0.118 (*p* < 0.001)	0.033 (*p* = 0.068; NS)
Cardiovascular diseases (other than HBP)	−0.034 (*p* = 0.061; NS)	−0.012 (*p* = 0.491; NS)	−0.012 (*p* = 0.512; NS)	−0.035 (*p* = 0.053; NS)
Respiratory diseases	0.072 (*p* < 0.001)	0.057 (*p* = 0.002)	0.068 (*p* < 0.001)	0.044 (*p* = 0.014)
Diabetes	0.009 (*p* = 0.626; NS)	0.011 (*p* = 0.552; NS)	0.017 (*p* = 0.351; NS)	−0.014 (*p* = 0.426; NS)
Rheumatic diseases	0.086 (*p* < 0.001)	0.071 (*p* < 0.001)	0.083 (*p* < 0.001)	0.047 (*p* = 0.008)
Central nervous system diseases	0.085 (*p* < 0.001)	0.094 (*p* < 0.001)	0.071 (*p* < 0.001)	0.034 (*p* = 0.06; NS)
Gastrointestinal diseases	0.038 (*p* = 0.034)	0.048 (*p* = 0.007)	0.037 (*p* = 0.038)	0.004 (*p* = 0.814; NS)
Endocrine diseases	0.012 (*p* = 0.515; NS)	0.034 (*p* = 0.058; NS)	−0.004 (*p* = 0.805; NS)	−0.005 (*p* = 0.798; NS)
Neoplasm	−0.052 (*p* = 0.004)	−0.024 (*p* = 0.179; NS)	−0.042 (*p* = 0.02)	−0.06 (*p* = 0.001)
Other diseases	0.039 (*p* = 0.031)	0.036 (*p* = 0.047)	0.024 (*p* = 0.187; NS)	0.019 (*p* = 0.279; NS)
**BP self-monitoring**	−0.184 (*p* < 0.001)	−0.123 (*p* < 0.001)	−0.147 (*p* < 0.001)	−0.16 (*p* < 0.001)
Normal BP during self-monitoring	−0.214 (*p* < 0.001)	−0.139 (*p* < 0.001)	−0.189 (*p* < 0.001)	−0.188 (*p* < 0.001)
**Frequency of adding salt in food**	0.254 (*p* < 0.001)	0.149 (*p* < 0.001)	0.214 (*p* < 0.001)	0.228 (*p* < 0.001)
**Frequency of reading PIL**	−0.239 (*p* < 0.001)	−0.177 (*p* < 0.001)	−0.17 (*p* < 0.001)	−0.207 (*p* < 0.001)
Dosage (element of interest from PIL)	−0.123 (*p* < 0.001)	−0.087 (*p* < 0.001)	−0.101 (*p* < 0.001)	−0.098 (*p* < 0.001)
Method of administration (element of interest from PIL)	−0.044 (*p* = 0.015)	−0.015 (*p* = 0.415; NS)	−0.03 (*p* = 0.09; NS)	−0.056 (*p* = 0.002)
Treatment duration (element of interest from PIL)	−0.026 (*p* = 0.15; NS)	−0.044 (*p* = 0.015)	−0.019 (*p* = 0.28; NS)	0.008 (*p* = 0.659; NS)
Adverse drug reactions (element of interest from PIL)	−0.126 (*p* < 0.001)	−0.095 (*p* < 0.001)	−0.092 (*p* < 0.001)	−0.101 (*p* < 0.001)
Dosage (pharmacist-related counselling)	−0.075 (*p* < 0.001)	−0.047 (*p* = 0.008)	−0.06 (*p* = 0.001)	−0.06 (*p* = 0.001)
Method of administration (pharmacist-related counselling)	−0.087 (*p* < 0.001)	−0.084 (*p* < 0.001)	−0.066 (*p* < 0.001)	−0.065 (*p* < 0.001)
Treatment duration (pharmacist-related counselling)	0.02 (*p* = 0.276; NS)	0.009 (*p* = 0.628; NS)	0.026 (*p* = 0.156; NS)	0.013 (*p* = 0.468; NS)
Adverse drug reactions (pharmacist-related counselling)	−0.04 (*p* = 0.028)	−0.036 (*p* = 0.046)	−0.032 (*p* = 0.075; NS)	−0.028 (*p* = 0.116; NS)

Legend: HBP = high blood pressure; PIL = patient information leaflets; and NS = non-significant.

**Table 3:** Validation results (average values and standard deviations) of the five machine learning models for each of the three quartile-based outcomes.

Validation Measure—Algorithm	Q1 (ARMS ≥ 15)	Q2 (ARMS ≥ 18)	Q3 (ARMS ≥ 22)
ROC AUC-LR	0.707 (±0.020)	0.700 (±0.016)	0.722 (±0.021)
**ROC AUC-CAT**	**0.728 (±0.023)**	**0.718 (±0.014)**	**0.734 (±0.022)**
ROC AUC-GBM	0.717 (±0.022)	0.706 (±0.016)	0.726 (±0.023)
ROC AUC-XGB	0.723 (±0.021)	0.714 (±0.019)	0.730 (±0.021)
ROC AUC-RF	0.713 (±0.026)	0.704 (±0.018)	0.724 (±0.023)
MCC-LR	0.245 (±0.035)	0.280 (±0.031)	0.290 (±0.027)
**MCC-CAT**	0.274 (±0.041)	**0.320 (±0.030)**	0.315 (±0.038)
MCC-GBM	0.273 (±0.037)	0.294 (±0.027)	0.311 (±0.040)
**MCC-XGB**	**0.279 (±0.041)**	0.312 (±0.025)	**0.317 (±0.041)**
MCC-RF	0.257 (±0.042)	0.290 (±0.031)	0.298 (±0.041)
ACCURACY-LR	0.650 (±0.019)	0.641 (±0.015)	0.660 (±0.015)
ACCURACY-CAT	0.721 (±0.031)	0.664 (±0.016)	0.687 (±0.024)
ACCURACY-GBM	0.692 (±0.031)	0.650 (±0.014)	0.674 (±0.022)
ACCURACY-XGB	0.724 (±0.030)	0.660 (±0.012)	0.684 (±0.022)
ACCURACY-VOTE	0.700 (±0.029)	0.647 (±0.016)	0.679 (±0.021)
PRECISION-LR	0.874 (±0.012)	0.703 (±0.016)	0.445 (±0.017)
PRECISION-CAT	0.863 (±0.016)	0.713 (±0.014)	0.475 (±0.031)
PRECISION-GBM	0.873 (±0.011)	0.705 (±0.014)	0.460 (±0.025)
PRECISION-XGB	0.864 (±0.013)	0.709 (±0.015)	0.471 (±0.028)
PRECISION-RF	0.863 (±0.011)	0.704 (±0.016)	0.464 (±0.027)
RECALL-LR	0.651 (±0.024)	0.637 (±0.024)	0.652 (±0.031)
RECALL-CAT	0.771 (±0.056)	0.682 (±0.032)	0.623 (±0.050)
RECALL-GBM	0.716 (±0.046)	0.660 (±0.024)	0.655 (±0.049)
RECALL-XGB	0.773 (±0.050)	0.684 (±0.028)	0.637 (±0.051)
RECALL-RF	0.737 (±0.041)	0.653 (±0.030)	0.611 (±0.045)

Legend: LR = Logistic Regression; CAT = CatBoost; GBM = Light GBM; XGB = XGBoost; RF = Random Forest; and MCC = Matthews Correlation Coefficient; the most important results are marked with bold.

**Table 4:** The importance of the predictive variables, computed through the permutation feature importance method for the first quartile-based outcome (Q1—ARMS ≥ 15).

Predictive Variable	Feature Importance—LR	Feature Importance—CAT	Feature Importance—GBM	Feature Importance—XGB	Feature Importance—RF
Age	4.26%	3.21%	4.57%	5.33%	2.82%
Sex	1.43%	1.03%	1.31%	1.11%	0.77%
Family support	0.16%	0.92%	0.69%	1.33%	0.50%
Level of education	2.04%	3.73%	2.72%	3.52%	1.56%
Number of daily administered drugs	1.81%	2.33%	4.39%	4.56%	1.05%
Number of daily administered supplements	7.75%	**12.76%**	15.17%	14.14%	18.50%
Cardiovascular diseases (other than HBP)	0.11%	2.58%	1.13%	1.66%	1.00%
Respiratory diseases	1.72%	1.32%	2.02%	1.43%	0.71%
Diabetes	0.32%	0.46%	0.09%	0.20%	0.14%
Rheumatic diseases	3.44%	2.42%	4.41%	4.11%	2.16%
Central nervous system diseases	7.46%	6.27%	7.38%	5.97%	3.74%
Gastrointestinal diseases	1.96%	2.71%	2.38%	2.47%	0.97%
Endocrine diseases	1.50%	0.89%	1.19%	1.08%	0.67%
Neoplasm	0.15%	0.07%	0.07%	0.07%	0.04%
Other diseases	0.75%	1.72%	2.93%	3.59%	1.03%
**BP self-monitoring**	5.53%	**6.53%**	5.35%	5.08%	7.47%
Normal BP during self-monitoring	22.48%	2.01%	5.18%	4.24%	4.21%
High BP during self-monitoring	5.94%	2.46%	0.65%	0.80%	2.95%
**Frequency of adding salt in food**	9.54%	**13.44%**	11.21%	10.61%	16.68%
**Frequency of reading PIL**	13.09%	**15.87%**	14.89%	14.73%	21.95%
Dosage (element of interest from PIL)	1.44%	0.80%	1.19%	1.45%	1.24%
Method of administration (element of interest from PIL)	0.54%	0.51%	0.57%	0.90%	0.37%
Treatment duration (element of interest from PIL)	0.13%	0.50%	0.26%	0.28%	0.21%
Adverse drug reactions (element of interest from PIL)	0.12%	1.66%	1.61%	2.23%	2.38%
Dosage (pharmacist-related counselling)	0.05%	2.35%	0.37%	0.82%	0.58%
Method of administration (pharmacist-related counselling)	4.98%	**8.09%**	6.56%	5.61%	5.11%
Treatment duration (pharmacist-related counselling)	0.83%	2.37%	1.01%	1.45%	0.91%
Adverse drug reactions (pharmacist-related counselling)	0.46%	0.98%	0.70%	1.22%	0.26%

Legend: LR = Logistic Regression; CAT = CatBoost; GBM = Light GBM; XGB = XGBoost; and RF = Random Forest; the most important results are marked with bold.

**Table 5:** The importance of the predictive variables, computed through the permutation feature importance method for the second quartile-based outcome (Q2—ARMS ≥ 18).

Predictive Variable	Feature Importance—LR	Feature Importance—CAT	Feature Importance—GBM	Feature Importance—XGB	Feature Importance—RF
Age	1.55%	2.39%	2.79%	3.86%	1.15%
Sex	0.24%	0.35%	0.32%	0.65%	0.24%
Family support	1.13%	0.79%	1.04%	1.36%	0.87%
Level of education	2.98%	3.93%	3.04%	3.32%	2.46%
Number of daily administered drugs	0.29%	1.64%	2.41%	2.78%	0.52%
Number of daily administered supplements	12.88%	**10.78%**	11.99%	11.59%	11.08%
Cardiovascular diseases (other than HBP)	0.06%	1.81%	0.72%	1.06%	0.27%
Respiratory diseases	1.58%	0.66%	1.93%	1.57%	0.88%
Diabetes	0.18%	0.19%	0.10%	0.29%	0.07%
Rheumatic diseases	4.09%	**4.92%**	6.95%	7.15%	6.23%
Central nervous system diseases	2.93%	2.25%	1.76%	1.72%	0.74%
Gastrointestinal diseases	0.75%	1.86%	1.18%	1.53%	0.52%
Endocrine diseases	0.02%	0.31%	0.13%	0.11%	0.14%
Neoplasm	0.80%	0.27%	0.34%	0.58%	0.30%
Other diseases	0.34%	1.63%	2.52%	2.66%	0.83%
**BP self-monitoring**	7.93%	**6.13%**	6.13%	5.67%	5.72%
Normal BP during self-monitoring	4.55%	2.88%	3.38%	3.12%	4.65%
High BP during self-monitoring	10.23%	4.16%	5.76%	3.84%	5.43%
**Frequency of adding salt in food**	28.89%	**27.20%**	28.24%	25.44%	37.22%
**Frequency of reading PIL**	9.61%	**11.17%**	9.15%	9.85%	10.36%
Dosage (element of interest from PIL)	3.66%	2.54%	3.34%	3.84%	3.16%
Method of administration (element of interest from PIL)	0.03%	0.52%	0.08%	0.32%	0.22%
Treatment duration (element of interest from PIL)	0.26%	1.19%	0.67%	0.61%	0.63%
Adverse drug reactions (element of interest from PIL)	0.09%	1.15%	0.77%	1.08%	0.92%
Dosage (pharmacist-related counselling)	0.43%	2.15%	0.68%	1.04%	0.63%
Method of administration (pharmacist-related counselling)	2.99%	3.10%	2.86%	2.81%	2.53%
Treatment duration (pharmacist-related counselling)	1.30%	3.18%	1.54%	1.66%	2.05%
Adverse drug reactions (pharmacist-related counselling)	0.20%	0.85%	0.21%	0.50%	0.19%

Legend: LR = Logistic Regression; CAT = CatBoost; GBM = Light GBM; XGB = XGBoost; and RF = Random Forest; the most important results are marked with bold.

**Table 6:** The importance of the predictive variables, computed through the permutation feature importance method for the third quartile-based outcome (Q3—ARMS ≥ 22).

Predictive Variable	Feature Importance—LR	Feature Importance—CAT	Feature Importance—GBM	Feature Importance—XGB	Feature Importance—RF
Age	0.24%	0.79%	0.69%	0.86%	0.38%
Sex	0.01%	0.15%	0.00%	0.06%	0.04%
Family support	1.01%	1.08%	2.13%	1.93%	1.41%
Level of education	3.99%	**7.66%**	6.89%	6.79%	5.53%
Number of daily administered drugs	1.50%	1.31%	1.45%	1.82%	1.42%
Number of daily administered supplements	2.26%	2.33%	2.20%	2.45%	1.29%
Cardiovascular diseases (other than HBP)	0.62%	1.16%	0.84%	0.98%	0.69%
Respiratory diseases	0.22%	0.23%	0.10%	0.21%	0.04%
Diabetes	0.03%	0.42%	0.05%	0.09%	0.05%
Rheumatic diseases	1.28%	1.22%	1.69%	1.65%	0.76%
Central nervous system diseases	0.52%	0.48%	0.31%	0.45%	0.16%
Gastrointestinal diseases	0.02%	0.75%	0.26%	0.40%	0.18%
Endocrine diseases	0.01%	0.06%	0.01%	0.02%	0.02%
Neoplasm	1.59%	1.44%	1.36%	1.75%	0.65%
Other diseases	0.22%	0.53%	0.62%	0.99%	0.49%
**BP self-monitoring**	4.42%	**6.55%**	5.44%	5.84%	7.44%
Normal BP during self-monitoring	5.70%	3.11%	0.62%	1.31%	4.59%
High BP during self-monitoring	30.51%	**6.33%**	14.05%	10.90%	8.50%
**Frequency of adding salt in food**	23.09%	**32.65%**	33.98%	31.75%	39.40%
**Frequency of reading PIL**	16.67%	**20.45%**	19.56%	19.74%	19.10%
Dosage (element of interest from PIL)	1.80%	0.78%	1.42%	1.37%	1.37%
Method of administration (element of interest from PIL)	0.22%	0.52%	0.27%	0.53%	0.55%
Treatment duration (element of interest from PIL)	2.22%	4.18%	3.47%	4.18%	2.64%
Adverse drug reactions (element of interest from PIL)	0.11%	0.58%	0.69%	0.91%	0.85%
Dosage (pharmacist-related counselling)	0.06%	1.32%	0.09%	0.45%	0.50%
Method of administration (pharmacist-related counselling)	1.34%	1.42%	1.25%	1.46%	0.97%
Treatment duration (pharmacist-related counselling)	0.30%	1.45%	0.42%	0.66%	0.48%
Adverse drug reactions (pharmacist-related counselling)	0.01%	1.05%	0.13%	0.44%	0.50%

Legend: LR = Logistic Regression; CAT = CatBoost; GBM = Light GBM; XGB = XGBoost; and RF = Random Forest; the most important results are marked with bold.

**Table 7:** The importance of the predictive variables, computed through the SHAP method (permutation algorithm) for CatBoost and all three quartile-based thresholds.

Predictive Variable	Feature Importance—Q1—ARMS ≥ 15	Feature Importance—Q2—ARMS ≥ 18	Feature Importance—Q3—ARMS ≥ 22
Age	3.42%	3.14%	2.32%
Sex	1.87%	1.20%	0.18%
Family support	1.48%	2.21%	2.81%
Level of education	4.59%	6.04%	6.14%
Number of daily administered drugs	2.55%	3.21%	3.28%
Number of daily administered supplements	9.58%	7.75%	3.81%
Cardiovascular diseases (other than HBP)	1.99%	1.67%	2.32%
Respiratory diseases	1.31%	2.16%	0.51%
Diabetes	0.70%	1.00%	0.16%
Rheumatic diseases	3.80%	4.21%	2.95%
Central nervous system diseases	3.30%	2.05%	1.07%
Gastrointestinal diseases	2.53%	2.11%	0.99%
Endocrine diseases	1.93%	0.64%	0.00%
Neoplasm	0.11%	0.45%	2.03%
Other diseases	2.35%	1.79%	1.19%
**BP self-monitoring**	8.02%	8.49%	8.41%
Normal BP during self-monitoring	5.57%	5.83%	3.19%
High BP during self-monitoring	4.42%	5.73%	10.28%
**Frequency of adding salt in food**	**11.02%**	**15.88%**	**20.52%**
**Frequency of reading PIL**	**11.00%**	**8.28%**	**13.55%**
Dosage (element of interest from PIL)	1.51%	2.51%	3.12%
Method of administration (element of interest from PIL)	1.32%	1.08%	1.45%
Treatment duration (element of interest from PIL)	1.03%	1.62%	4.24%
Adverse drug reactions (element of interest from PIL)	2.69%	1.55%	1.18%
Dosage (pharmacist-related counselling)	2.41%	1.92%	1.02%
Method of administration (pharmacist-related counselling)	5.41%	3.36%	2.59%
Treatment duration (pharmacist-related counselling)	2.28%	2.42%	0.54%
Adverse drug reactions (pharmacist-related counselling)	1.81%	1.70%	0.15%

The most important results are marked with bold.

## Data Availability

The original contributions presented in this study are included in the article. Further inquiries can be directed to the corresponding author.

## Abbreviations

The following abbreviations are used in this manuscript:

ARMS	Adherence to Refills and Medications Scale
HPB	High blood pressure
PILs	Patient information leaflets
SHAP	SHapley Additive exPlanations
